# iNOS/Arg-1 balance and sIgA levels are associated with intestinal mucosal immune defense in children chronically exposed to air pollutants and lead

**DOI:** 10.3389/fimmu.2026.1903043

**Published:** 2026-08-28

**Authors:** Jingle Qiu, Xia Huo, Yu Huang, Binghui Liu, Tengyang Huang, Xijin Xu

**Affiliations:** 1 Laboratory of Environmental Medicine and Developmental Toxicology, Shantou University Medical College, Shantou, Guangdong, China; 2 Laboratory of Environmental Medicine and Developmental Toxicology, Guangdong Key Laboratory of Environmental Pollution and Health, School of Environment and Climate, Jinan University, Guangzhou, Guangdong, China; 3 Department of Cell Biology and Genetics, Shantou University Medical College, Shantou, Guangdong, China

**Keywords:** co-exposure, e-waste recycling area, iNOS/Arg-1 ratio, intestinal mucosal immunity, macrophage polarization, network toxicology, SIgA

## Abstract

**Background:**

Air pollutants and lead (Pb) are widespread environmental risk factors that may disrupt children’s intestinal immune homeostasis. However, the immunoregulatory responses of intestinal mucosal immunity to chronic co-exposure remain poorly understood.

**Methods:**

We conducted a cross-sectional study of 238 children aged 3–6 years from an e-waste recycling area (Guiyu) and a reference area (Haojiang). Estimated average daily doses (ADDs) of air pollutants and blood Pb levels were assessed. Peripheral blood cells, serum cytokines, macrophage polarization markers, including inducible nitric oxide synthase (iNOS) and arginase-1 (Arg-1), and serum secretory immunoglobulin A (sIgA) were measured. Single-pollutant regression, Bayesian kernel machine regression, and restricted cubic spline analyses were performed. Network toxicology was used to explore potential molecular targets and immune-related pathways.

**Results:**

Children from Guiyu had higher blood Pb levels and higher ADDs of PM_2.5_, PM_10_, NO_2_, SO_2_, and CO. They also showed increased lymphocyte and monocyte counts, decreased iNOS, increased Arg-1 and IL-4, and a lower iNOS/Arg-1 ratio. Mixed exposure was inversely associated with iNOS/Arg-1 ratio. Serum sIgA levels did not differ significantly between areas, and mixed exposure was not directly associated with serum sIgA; however, a nonlinear association was observed between the iNOS/Arg-1 ratio and serum sIgA. Network toxicology implicated inflammatory signaling, oxidative stress, nitric oxide biosynthesis, arginine metabolism, and IgA-related intestinal immune pathways.

**Conclusions:**

Long-term co-exposure to air pollutants and Pb was associated with a lower iNOS/Arg-1 ratio and an M2-like macrophage polarization-related marker profile in children. The observed nonlinear association between the iNOS/Arg-1 ratio and sIgA may reflect a potential relationship between macrophage polarization-related immune responses and sIgA-associated mucosal immunity. These findings highlight pollutant-associated immune alterations related to intestinal mucosal defense.

## Graphical Abstract

**Figure:**
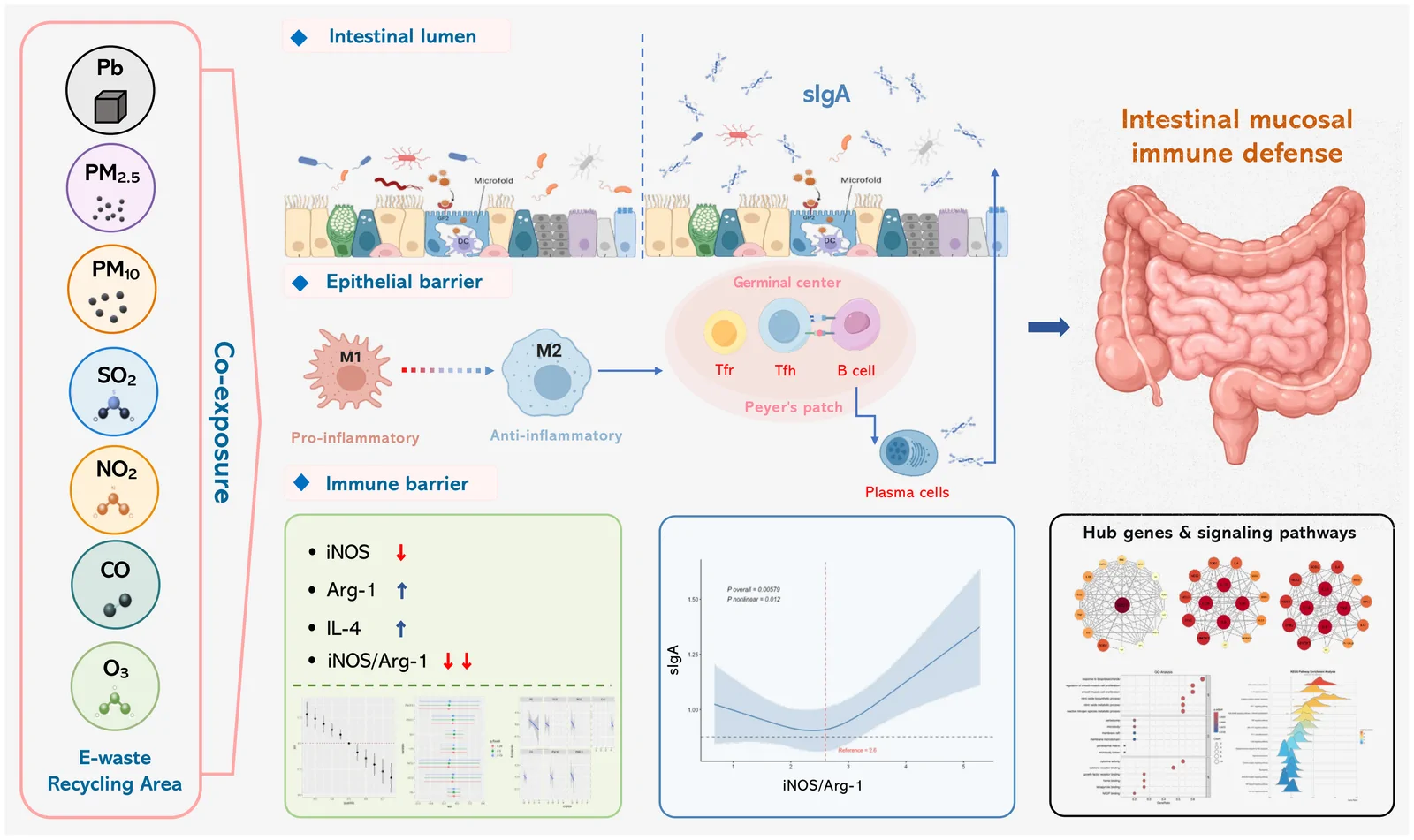

## 1 Introduction

Rapid global industrialization has elevated air pollution to a major public health concern, with particularly pronounced effects on children as a sensitive population (1). Air pollutants typically do not exist in isolation but form complex mixtures with toxic heavy metals such as lead (Pb) (2). As a persistent environmental contaminant, Pb can cause long-term adverse effects on children’s immune, nervous, and digestive systems, even at relatively low exposure levels (3–5). Our previous studies have shown that Pb and air pollutants are associated with intestinal mucosal injury, increased intestinal permeability, gut microbial disturbance, and enteritis-related outcomes in children, suggesting that the intestine may be an important target organ under mixed environmental exposure (6–10).

The intestine serves as both the primary site for nutrient absorption and an important immune organ, maintaining tolerance to commensal microbiota while mounting effective defenses against pathogens (11, 12). Macrophage-related immune regulation plays a central role in intestinal homeostasis. Classically activated M1 macrophage responses are commonly characterized by pro-inflammatory and inducible nitric oxide synthase (iNOS)-related antimicrobial activity, whereas alternatively activated M2-associated responses are linked to arginase-1 (Arg-1) expression, anti-inflammatory activity, and tissue repair. The M1/M2 balance is closely involved in intestinal inflammation and mucosal barrier homeostasis (13, 14). In parallel, secretory immunoglobulin A (sIgA) is an important component of mucosal immune defense, contributing to immune exclusion by binding pathogens and toxins and limiting their contact with the intestinal epithelium (15). Thus, the iNOS/Arg-1 balance and sIgA may serve as immune-related indicators in evaluating pollutant-associated intestinal immune alterations.

Environmental pollutants may influence intestinal immunity not only by inducing mucosal injury but also by reshaping immune defense and regulatory responses under sustained environmental stress (16–18). Immune responses to chronic low-dose exposure may differ substantially from those induced by acute high-dose exposure, potentially involving compensatory or tolerance-related mechanisms (19–21). However, whether long-term co-exposure to mixed air pollutants and Pb is associated with altered intestinal mucosal immune regulation in children remains unclear.

Although previous studies have investigated the immunotoxic effects of Pb or air pollutants separately (22, 23), several knowledge gaps remain regarding intestinal mucosal immune regulation in children under chronic co-exposure. First, it remains unclear whether children from polluted environments exhibit altered iNOS/Arg-1 balance, reflecting a potential shift in macrophage-related functional states. Second, the relationship between pollutant exposure and serum sIgA, an immune-related indicator linked to mucosal defense, has not been fully characterized. Third, most existing studies have focused on single pollutants, whereas children in e-waste areas are exposed to complex pollutant mixtures with potential joint or interactive effects.

To address these gaps, this study builds on our previous findings of pollutant-associated intestinal mucosal damage in children from an e-waste area. We assessed air pollutant exposure, blood Pb levels, iNOS, Arg-1, serum cytokines, peripheral immune cells, and serum sIgA, and applied single-pollutant regression, restricted cubic spline analysis, Bayesian kernel machine regression, and network toxicology to evaluate individual, mixture-related, nonlinear, and pathway-level associations. This study aimed to examine whether chronic co-exposure to air pollutants and Pb is associated with intestinal immune defense in children, with a focus on the iNOS/Arg-1 balance, serum sIgA, and their potential relationship. These findings may provide immunological evidence for understanding early-life intestinal mucosal immune alterations in polluted environments.

## 2 Materials and methods

### 2.1 Study participants

A cross-sectional observational study was conducted to investigate the associations of environmental pollutant exposure with intestinal mucosal immunity and macrophage polarization-related markers in children. Participants were recruited from two kindergartens located in Guiyu Town (the e-waste recycling area) and Haojiang District (the reference area), Shantou City, Guangdong Province, China, between November and December 2018. Guiyu and Haojiang are two towns in Shantou City, with Haojiang located approximately 31.6 km east of Guiyu. Apart from the absence of e-waste recycling activities in Haojiang, the two areas are comparable in terms of ethnicity, cultural background, and socioeconomic status (24, 25). Eligible participants were children aged 3–6 years who had resided in the study area for at least one year.

A total of 680 children completed the questionnaire survey. Of these, 97 children declined to participate in the health examination, leaving 583 children for physical examination and biological sample collection. After excluding children with incomplete information, evidence of inflammation, recent illness, antibiotic use within the previous 3 months, or residence in the study area for less than one year, 454 eligible children remained. Because sufficient blood volume was required to complete all laboratory analyses, 238 age- and sex-matched children (121 from Guiyu and 117 from Haojiang) were finally included in the present study. The participant selection process is shown in **Supplementary Figure 1**.

All participants underwent a standardized physical examination, and fasting venous blood and urine samples were collected. The study was approved by the Medical Ethics Committee of Shantou University Medical College, and written informed consent was obtained from the legal guardians of all participants before enrollment.

### 2.2 Assessment of air pollutant exposure

Consistent with previous methodologies, annual air quality data for 2018 were acquired for Haojiang District and Chaonan District (Guiyu region) in Shantou City via the China National Urban Air Quality Real-time Release Platform (https://air.cnemc.cn:18007/). The data included concentrations of nitrogen dioxide (NO_2_), sulfur dioxide (SO_2_), carbon monoxide (CO), ozone (O_3_), inhalable particulate matter (PM_10_), and fine particulate matter (PM_2.5_). According to the participants’ residential addresses, kindergarten locations, and the geographic locations of the corresponding air quality monitoring stations, all participants resided within an 8-km radius of their respective monitoring station (26).

The air quality comprehensive index (AQCI) for each contaminant was calculated with the following formula:


AQCI=Ci/Si

Where *Ci* denotes the measured concentration of the target pollutant, and *Si* refers to the Class II standard value specified in China’s Ambient Air Quality Standard (27–29).

To estimate inhalation-related exposure to the six air pollutants, an average daily dose (ADD) metric was calculated based on the annual AQCI values and child-specific parameters, including body weight and time spent outdoors, using the following formula:


ADD=(AQCI×IR)/Weight

Where *IR* represents inhalation rate, which was adjusted according to daily outdoor activity levels and individual body weight. Because pollutant concentrations were assigned at the study-area level, whereas body weight and outdoor activity time varied among children, the calculated ADD should be interpreted as an area-level exposure metric modified by child-specific inhalation-related characteristics rather than as a residentially resolved or directly measured individual exposure dose.

### 2.3 Measurement of blood Pb levels

Whole blood Pb levels were measured using graphite furnace atomic absorption spectrometry GF-AAS (ZEEnit 650, Analytik Jena, Germany) with a method from a previous study (30). The temperature program was set as follows: drying at 90 °C, 105 °C, and 110 °C, ashing at 800 °C, and atomization at 1500 °C. The method showed recoveries of 91.06–101.20% from spiked blood samples analyzed in triplicate, with detailed analytical procedures described elsewhere (31). The limit of detection (LOD) was determined to be 0.084 μg/dL based on measurements of a 0.5% nitric acid blank. A five-point calibration curve was applied before sample analysis, with a correlation coefficient exceeding 0.99. All samples were analyzed in duplicate, and the mean values were reported; relative standard deviations (RSD) below 15% were considered acceptable.

### 2.4 Measurement of peripheral blood cells and cytokines

Serum concentrations of IL-1β, TNF-α, and IL-4 were quantified using multiplex enzyme-linked immunosorbent assay (ELISA) kits (Thermo Fisher Scientific, USA). Meanwhile, peripheral blood monocytes and lymphocytes were enumerated with an automated hematology analyzer (Sysmex XE-2100, Japan).

### 2.5 Measurement of iNOS, Arg-1, and sIgA

Serum iNOS levels were quantified using a Human iNOS ELISA Kit (EEL035, Thermo Fisher Scientific, USA). Serum Arg-1 concentrations were measured with a Human Arginase-1 ELISA Kit (BMS2216, Thermo Fisher Scientific, USA). sIgA levels in serum were determined using a Secretory IgA (Human) ELISA Kit (KA3980, Abnova, Taiwan). All assays were performed in accordance with the manufacturer’s protocols.

### 2.6 Network toxicology

Targets associated with the pollutants (Pb, SO_2_, NO_2_, CO, O_3_, PM_10_, and PM_2.5_) were retrieved from the Comparative Toxicogenomics Database (CTD, https://ctdbase.org/). Enteritis-related targets were obtained from CTD and the GeneCards database (https://www.genecards.org/). Overlapping targets between pollutant-related and intestinal inflammation-related genes were identified using the “VennDiagram” package in R software. These common targets were then imported into the STRING database (https://string-db.org/) to construct a protein-protein interaction (PPI) network, with the species set to Homo sapiens and a confidence threshold of 0.4. Next, Cytoscape software (version 3.9.1) together with the Centiscape 2.2 plugin was used to screen for hub genes based on three topological metrics: betweenness centrality, closeness centrality, and degree centrality. Finally, Gene Ontology (GO) functional enrichment analysis and Kyoto Encyclopedia of Genes and Genomes (KEGG) pathway enrichment analysis were performed on the core targets using the R package clusterProfiler to identify potentially relevant biological processes and pathways through which environmental pollutant exposure affects intestinal health.

### 2.7 Statistical analysis

Data completeness was assessed before statistical analyses. Only one participant had a missing value for breastfeeding duration. This value was imputed using the most frequent category (mode imputation) to maintain a consistent denominator in the descriptive analyses. No other missing values required imputation.

All statistical tests were two-sided, and statistical significance was set at *P* < 0.05. Continuous variables were presented as mean ± standard deviation (SD) or median with interquartile range (IQR), while categorical variables were summarized as frequencies and percentages. Comparisons between the Haojiang and Guiyu cohorts were performed using independent t-tests (for normally distributed continuous variables), Mann–Whitney U tests (for non-normally distributed variables), or chi-square tests (for categorical variables). Spearman’s rank correlation analysis was used to assess correlations among environmental pollutants.

Because blood Pb concentrations, air pollutant concentrations, the iNOS/Arg-1 ratio, and serum sIgA levels exhibited positively skewed distributions, these variables were natural log-transformed before regression, Bayesian kernel machine regression (BKMR), and restricted cubic spline (RCS) analyses to improve distributional normality and satisfy model assumptions. Regression coefficients are presented based on the ln-transformed variables.

For single-pollutant exposure analysis, linear regression models were applied to evaluate the associations between each pollutant and the iNOS/Arg-1 as well as sIgA levels, with results reported as *β* coefficients and 95% confidence intervals (CI). RCS models with three knots were employed to explore potential non-linear dose–response relationships between pollutant exposure and outcomes (iNOS/Arg-1 and sIgA), as well as between the iNOS/Arg-1 and sIgA.

For mixed-exposure analysis, BKMR was used to evaluate the joint effects of multi-pollutant exposure on the iNOS/Arg-1 ratio and serum sIgA. Separate Gaussian BKMR models were fitted for each outcome using 5,000 MCMC iterations with component-wise variable selection (varsel = TRUE) and Gaussian kernel estimation (est.h = TRUE). Model convergence was assessed by visual inspection of trace plots for the regression coefficients (*β*), residual variance (*σ²*), and kernel parameters (*r*). Overall mixture effects, univariate exposure–response functions, conditional single-pollutant effect estimates, and pairwise interaction patterns among pollutants were obtained from the fitted BKMR models. Pairwise interaction patterns were summarized using the BKMR-derived interaction matrix.

All statistical analyses were performed using R version 4.4.2, along with statistical software including Prism 10.1.1 (GraphPad) and SPSS 27.0 (IBM). Figures were generated using R (ggplot2 package) and GraphPad Prism.

### 2.8 Covariates

Based on causal inference principles, potential confounders were identified using a directed acyclic graph (DAG) constructed with DAGitty. Candidate variables were first selected according to prior biological knowledge and published literature and were then evaluated within the DAG framework to derive a minimally sufficient adjustment set (**Supplementary Figure 2**). The final adjustment set included sex, age, pencil/eraser biting habit, direct contact with e-waste, passive smoking, breastfeeding duration, probiotic use within the previous 3 months, air conditioning use with closed windows, and residential e-waste pollution within 50 m. Food-related diarrhea and growth-related measures, including BMI and chest circumference, were not included because they may represent downstream consequences of exposure and their adjustment could introduce overadjustment bias.

## 3 Results

### 3.1 Demographic characteristics

The final analytical sample comprised 238 children, including 117 from the Haojiang district and 121 from the Guiyu district (**Table 1**). The two groups were comparable in age and sex, with no statistically significant differences observed for either variable (both *P* > 0.05). However, children in the Haojiang cohort had a significantly higher BMI than those in the Guiyu cohort (15.61 ± 1.17 vs. 14.78 ± 1.66 kg/m², *P* < 0.001), whereas chest circumference did not differ significantly between the two groups (52.83 ± 3.24 vs. 52.08 ± 3.10 cm, *P* = 0.070). Only 11.1% of children in the Haojiang cohort reported occasional contact with e-waste, compared with 28.1% in the Guiyu cohort (*P* < 0.001). Similarly, e-waste pollution within 50 m of the residence was reported for 2.6% of children in the Haojiang cohort and 22.3% in the Guiyu cohort (*P* < 0.001). No significant between-group differences were observed in probiotic use during the previous 3 months or in the use of air conditioning with windows closed (both *P* > 0.05), whereas passive smoking exposure differed significantly between the two groups (*P* = 0.021). Breastfeeding duration also differed significantly between the two cohorts (*P* < 0.001).

**Table 1:** Demographic characteristics of the study population.

Characteristics	Haojiang (n=117)	Guiyu (n=121)	Statistics	*P*-value
Sex (male/female)	69/48	67/54	χ^2^ = 0.315	0.574^b^
Age (years) (mean ± SD)	4.91 ± 0.94	5.04 ± 0.88	t = -1.143	0.254^a^
BMI (kg/m^2^) (mean ± SD)	15.61 ± 1.17	14.78 ± 1.66	t = 4.454	< 0.001^a^
Chest circumference (cm) (mean ± SD)	52.83 ± 3.24	52.08 ± 3.10	t = 1.819	0.070
Probiotic use in the past 3 months [n (%)]			χ^2^ = 0.319	0.572^b^
No	107 (91.5)	113 (93.4)		
Yes	10 (8.5)	8 (6.6)		
Passive smoking			χ^2^ = 11.563	0.021^b^
None	60 (51.3)	42 (34.7)		
2 cigarettes/day	12 (10.3)	24 (19.8)		
Half pack/day	15 (12.8)	21 (17.4)		
One pack/day	26 (22.2)	23 (19.0)		
>1 pack/day	4 (3.4)	11 (9.1)		
Contact with electronic waste [n (%)]			χ^2^ = 10.832	< 0.001^b^
Never	104 (88.9)	87 (71.9)		
Occasionally	13 (11.1)	34 (28.1)		
E-waste pollution within 50 m of residence [n (%)]			χ^2^ = 21.062	< 0.001^b^
Yes	3 (2.6)	27 (22.3)		
No	114 (97.4)	94 (77.7)		
Air conditioning use with closed windows [n (%)]			χ^2^ = 3.258	0.071^b^
Yes	29 (24.8)	43 (35.5)		
No	88 (75.2)	78 (64.5)		
Breastfeeding duration [n (%)]			χ^2^ = 41.129	< 0.001^b^
None	12 (10.3)	46 (38.0)		
0–6 months	35 (29.9)	45 (37.2)		
6–12 months	50 (42.7)	27 (22.3)		
12–18 months	18 (15.4)	2 (1.7)		
>18 months	2 (1.7)	1 (0.8)		
Frequency of diarrhea [n (%)]				
Never	98 (83.8)	82 (67.8)	χ^2^ = 8.254	0.004^b^
1–2 times per month	19 (16.2)	39 (32.2)		
Visit hospital for digestive disorders [n (%)]			χ^2^ = 6.193	0.013^b^
Yes	20 (17.1)	8 (6.7)		
No	97 (82.9)	113 (93.3)		

BMI, body mass index.

*P* < 0.05 is considered statistically significant.

a Analysis by independent-sample t-test.

b Analysis by Pearson chi-square test.

Regarding gastrointestinal symptoms, the Guiyu cohort exhibited a significantly higher frequency of diarrhea than the Haojiang cohort (*χ²* = 8.254, *P* = 0.004). Specifically, 32.2% of children in the Guiyu cohort experienced diarrhea 1–2 times per month, compared with 16.2% of children in the Haojiang cohort. In contrast, the proportion of children who had visited a hospital for digestive disorders was significantly higher in the Haojiang cohort than in the Guiyu cohort (17.1% vs. 6.7%, *P* = 0.013). Collectively, these findings indicate that children in the Guiyu cohort experienced greater e-waste exposure and a higher frequency of diarrhea, whereas healthcare utilization for digestive disorders was more common among children in the Haojiang cohort.

### 3.2 Pollutant distribution and correlation in children

**Table 2** displays the levels of environmental pollutant exposure and the calculated ADD for both groups of children. The findings indicated that children in Guiyu had markedly higher levels of several air pollutants than children in Haojiang. More specifically, when set against the Haojiang cohort, the Guiyu cohort had substantially increased levels of SO_2_ (13.00 vs. 9.00 μg/m³), NO_2_ (20.00 vs. 12.00 μg/m³), CO (700.00 vs. 600.00 μg/m³), PM_10_ (47.00 vs. 37.00 μg/m³), and PM_2.5_ (25.00 vs. 19.00 μg/m³), with all comparisons reaching *P* < 0.001. In contrast, O_3_ levels were markedly reduced in the Guiyu cohort relative to the Haojiang cohort (67.00 vs. 74.00 μg/m³, *P* < 0.001).

**Table 2:** Comparison of ambient air pollutant concentrations, average daily dose, and blood Pb levels between Haojiang and Guiyu.

Pollutant	Haojiang	Guiyu	Statistics	*P*-value
Ambient air concentrations				
SO_2_ (μg/m³) [median (P25, P75)]	9.00 (8.00, 11.00)	13.00 (10.00, 19.00)	Z = 59.846	< 0.001^b^
NO_2_ (μg/m³) [median (P25, P75)]	12.00 (8.00, 18.00)	20.00 (14.00, 26.00)	Z = 52.590	< 0.001^b^
CO (μg/m³) [median (P25, P75)]	600.00 (600.00, 800.00)	700.00 (600.00, 800.00)	Z = 15.586	< 0.001^b^
O_3_ (μg/m³) [median (P25, P75)]	74.00 (49.00, 104.00)	67.00 (43.00, 101.00)	Z = 8.911	< 0.001^b^
PM_10_ (μg/m³) [median (P25, P75)]	37.00 (26.00, 51.00)	47.00 (33.00, 63.00)	Z = 27.184	< 0.001^b^
PM_2.5_ (μg/m³) [median (P25, P75)]	19.00 (14.00, 28.00)	25.00 (18.00, 37.00)	Z = 30.851	< 0.001^b^
Average daily dose				
SO_2_ [m^3^/(day·kg)] (mean ± SD)	0.07 ± 0.01	0.14 ± 0.02	t = -29.105	< 0.001^a^
NO_2_ [m^3^/(day·kg)] (mean ± SD)	0.16 ± 0.02	0.26 ± 0.04	t = -22.910	< 0.001^a^
CO [m^3^/(day·kg)] (mean ± SD)	0.08 ± 0.01	0.09 ± 0.01	t = -5.842	< 0.001^a^
O_3_ [m^3^/(day·kg)] (mean ± SD)	0.22 ± 0.03	0.23 ± 0.04	t = -1.400	0.163 ^a^
PM_10_ [m^3^/(day·kg)] (mean ± SD)	0.52 ± 0.08	0.73 ± 0.12	t = -16.010	< 0.001^a^
PM_2.5_ [m^3^/(day·kg)] (mean ± SD)	0.14 ± 0.02	0.21 ± 0.04	t = -19.387	< 0.001^a^
Blood Pb levels				
Pb (μg/dL) [median (P25, P75)]	4.06 (3.27, 4.90)	4.60 (3.72, 5.96)	Z = 3.555	< 0.001^b^

SD, standard deviation; Pb, lead.

a Analysis by independent-sample t-test.

b Analysis by Mann-Whitney U test.

Regarding ADD assessment, the Guiyu cohort displayed markedly higher values for SO_2_ [0.14 vs. 0.07 m³/(day·kg)], NO_2_ [0.26 vs. 0.16 m³/(day·kg)], CO [0.09 vs. 0.08 m³/(day·kg)], PM_10_ [0.73 vs. 0.52 m³/(day·kg)], and PM_2.5_ [0.21 vs. 0.14 m³/(day·kg)] than the Haojiang group, whereas the two groups showed no marked disparity in their ADD for O_3_ (0.23 vs. 0.22 m³/(day·kg), *P* = 0.163). Furthermore, markedly higher blood Pb levels were observed in the Guiyu cohort relative to the Haojiang group (4.60 vs. 4.06 μg/dL, *P* < 0.001). Collectively, these findings indicate that children in the Guiyu group had higher exposure levels and doses of multiple environmental pollutants than those in the Haojiang group, particularly for SO_2_, NO_2_, PM_2.5_, PM_10_, and blood Pb, indicating that children residing in the Guiyu region are at greater risk of being exposed to environmental pollutants.

**Figure 1:**
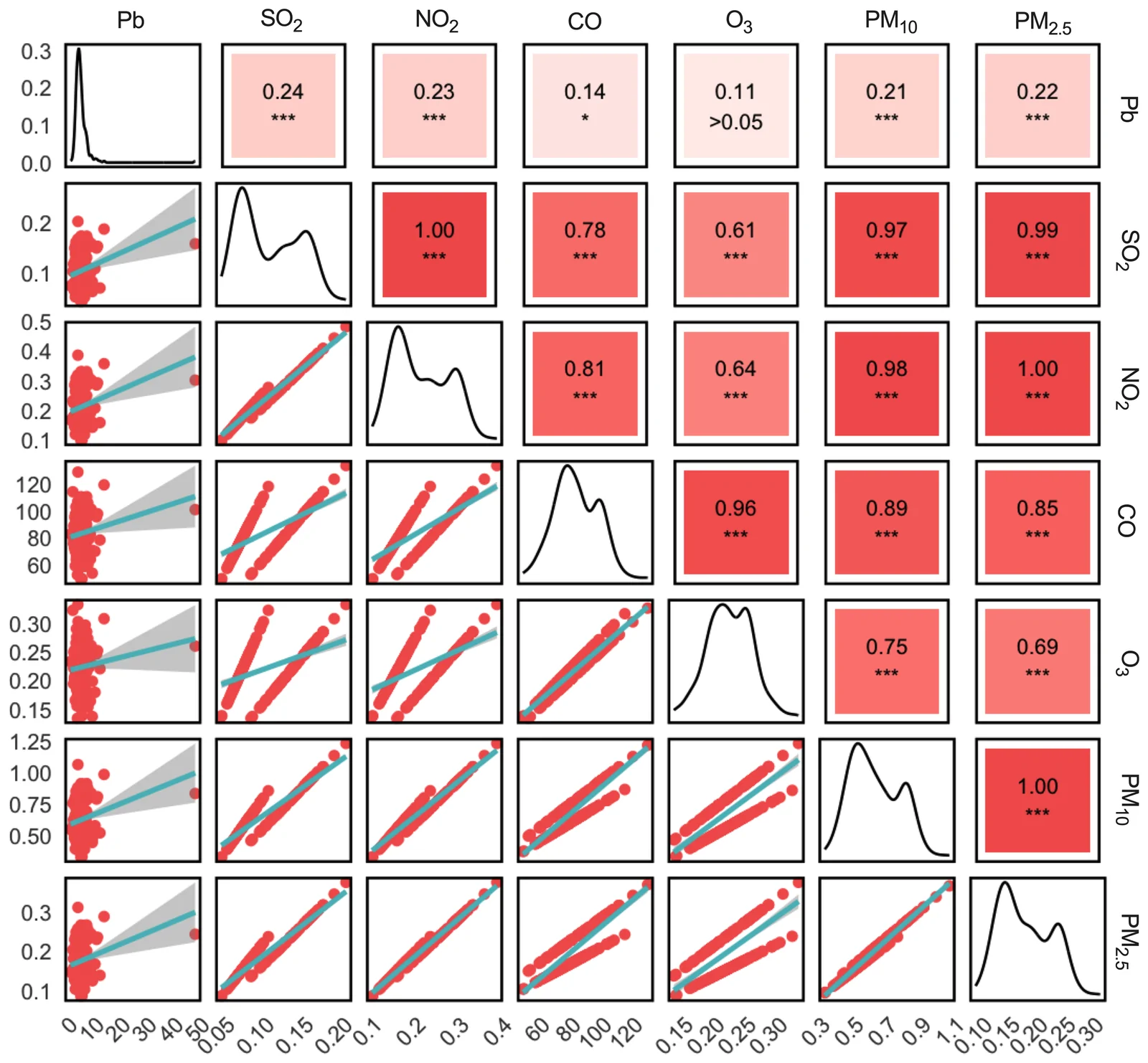
Spearman correlation heatmap of associations between air pollutants ADD and blood Pb levels. *P < 0.05, ***P < 0.001 indicate statistical significance.

Spearman correlation analysis (**Figure 1**) revealed that air pollutants (SO_2_, NO_2_, CO, O_3_, PM_10_, PM_2.5_) were strongly and positively correlated with one another, with coefficients in the range of 0.61–1.00 (*P* < 0.001), suggesting high collinearity and common origins. Pb showed weak but statistically significant positive correlations with most air pollutants (*r* = 0.14–0.24, *P* < 0.05), except for O_3_, which showed no significant association with Pb (*P* > 0.05).

### 3.3 Peripheral blood cells, cytokines, macrophage polarization markers, and sIgA

**Figure 2:**
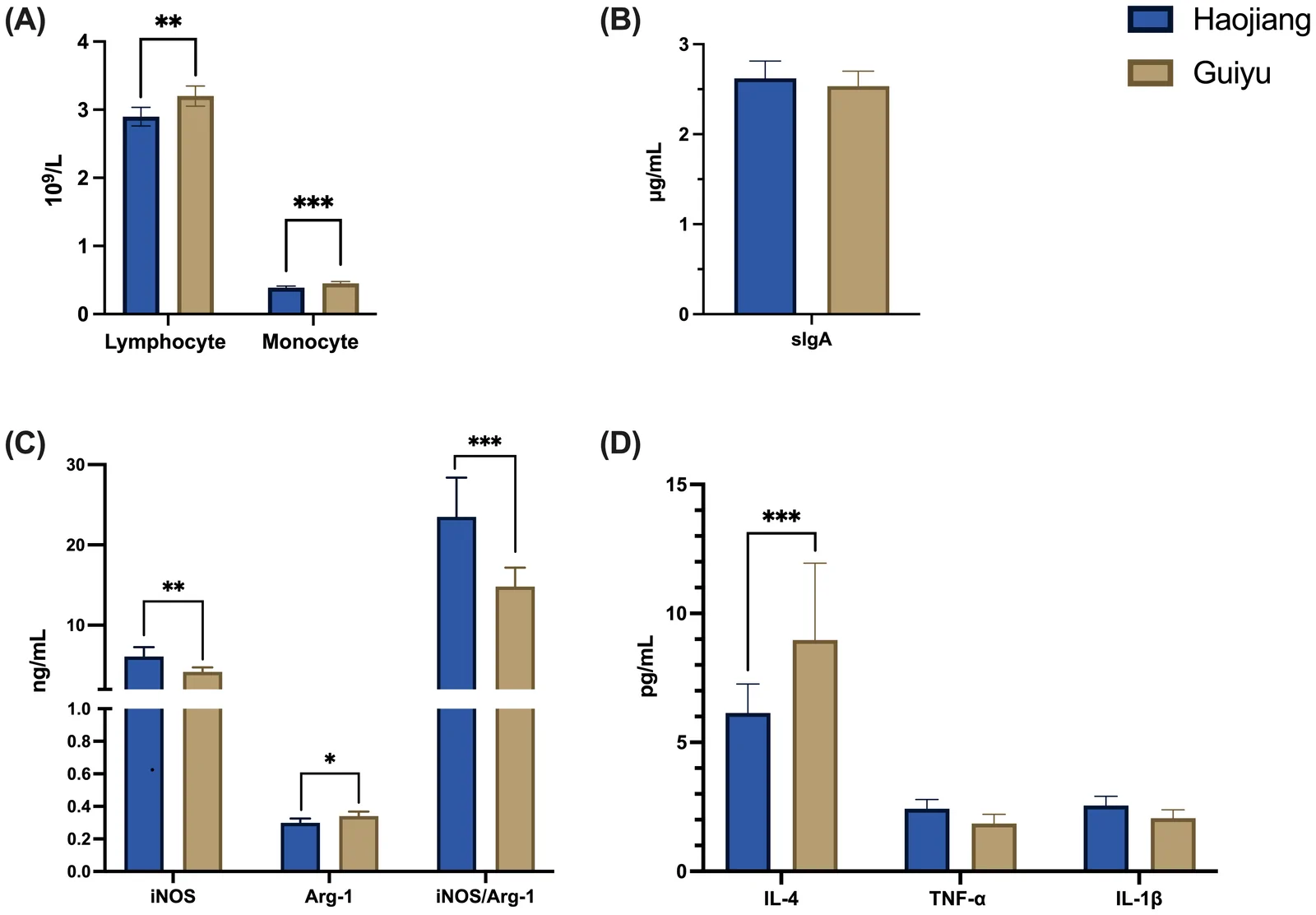
Comparison of immune and inflammatory markers between children in Haojiang and Guiyu. **(A)** Peripheral blood lymphocyte and monocyte counts. **(B)** Serum sIgA levels. **(C)** Serum levels of iNOS, Arg-1, and iNOS/Arg-1 ratio. **(D)** Serum levels of inflammatory cytokines IL-4, TNF-α, and IL-1β. Data are presented as mean ± standard error (SE). *P < 0.05, **P < 0.01, ***P < 0.001 indicate statistical significance.

**Figure 2** compares the Guiyu and Haojiang cohorts with respect to peripheral blood cell counts (**Figure 2A**), sIgA levels (**Figure 2B**), macrophage polarization markers (**Figure 2C**), and cytokine levels (**Figure 2D**). The results showed that lymphocyte and monocyte counts were markedly elevated among the Guiyu cohort compared with the Haojiang cohort (lymphocytes: 3.20 vs. 2.90 × 10^9^/L, *P* = 0.005; monocytes: 0.45 vs. 0.39 × 10^9^/L, *P* < 0.001). Meanwhile, no marked disparities were found in serum IL-1β or TNF-α levels across the two cohorts (both *P* > 0.05). With respect to macrophage polarization markers, markedly reduced serum iNOS levels were observed in the Guiyu cohort relative to the Haojiang group (3.18 vs. 5.06, *P* = 0.011), whereas the M2-type markers IL-4 (7.59 vs. 3.59, *P* = 0.001) and Arg-1 (0.32 vs. 0.29, *P* = 0.008) were both markedly elevated in the Guiyu cohort. The iNOS/Arg-1 ratio was calculated as a summary indicator of the relative balance between M1- and M2-associated markers, and the results showed that this ratio was markedly reduced in the Guiyu cohort relative to the Haojiang cohort (11.17 vs. 16.03, *P* < 0.001). In addition, no marked disparity in serum sIgA levels was observed between the two groups (control median: 2.46 μg/mL; exposed median: 2.41 μg/mL). Collectively, these findings indicate a macrophage polarization-related marker profile characterized by lower iNOS, higher Arg-1 and IL-4, and a reduced iNOS/Arg-1 ratio, suggesting a tendency toward M2-associated features in Guiyu children.

### 3.4 Associations of individual air pollutants and Pb exposure with iNOS/Arg-1 and sIgA

**Figure 3:**
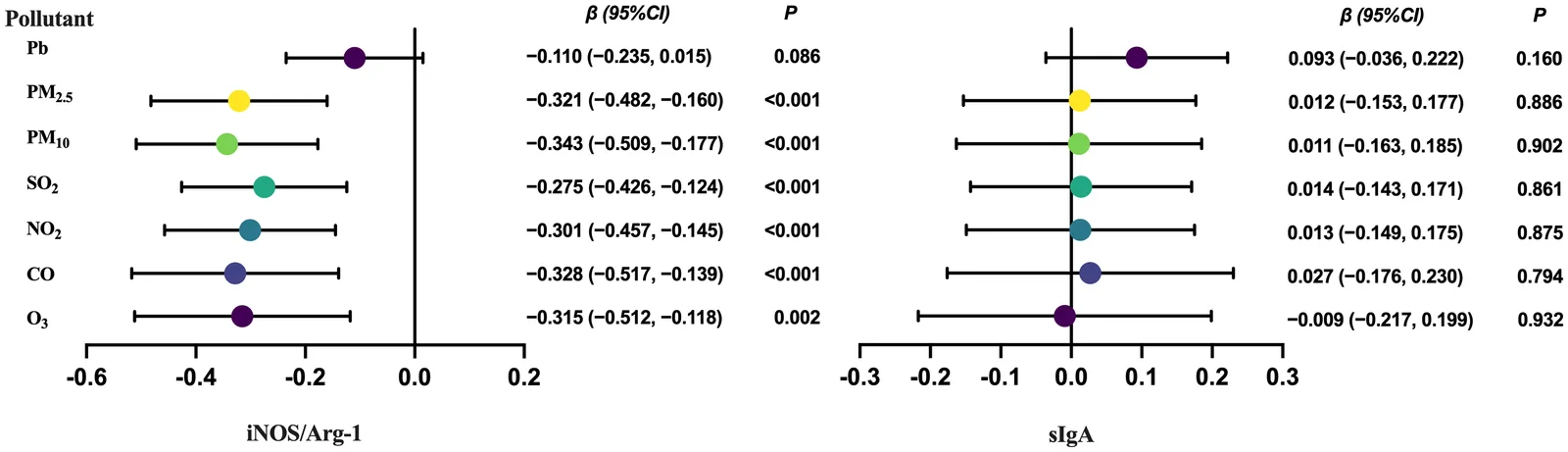
Associations of environmental pollutant exposures with iNOS/Arg-1 and sIgA. Multivariable linear regression models were adjusted for sex, age, pencil/eraser biting habit, probiotic use in the past 3 months, history of e-waste exposure, passive smoking, breastfeeding duration, air conditioning use with closed windows, and e-waste pollution within 50m of residence. *β* coefficients with 95% confidence intervals (CI) and corresponding *P*-values are presented.

The associations between the ADD of ambient air pollutants or blood Pb levels and the iNOS/Arg-1 ratio as well as sIgA are presented in **Figure 3**. Blood Pb levels showed an inverse association with the iNOS/Arg-1 ratio; however, the association did not reach statistical significance after adjustment for potential confounders (*β* = −0.110, 95% CI: −0.235 to 0.015, *P* = 0.086). In contrast, all ambient air pollutants were significantly negatively associated with the iNOS/Arg-1 ratio, with standardized *β* coefficients ranging from −0.275 to −0.343 (all *P* ≤ 0.002). Regarding serum sIgA, neither blood Pb nor any of the ambient air pollutants showed statistically significant associations with serum sIgA levels in children (all *P* > 0.05).

We further performed RCS analyses (**Supplementary Figures 3**, **4**) to investigate potential non-linear dose–response associations of pollutant exposures with both the iNOS/Arg-1 ratio and sIgA. No significant non-linear associations were observed between any of the pollutants and either the iNOS/Arg-1 ratio or sIgA (all *P* > 0.05).

### 3.5 Mixed effects of air pollutants and Pb exposure on iNOS/Arg-1 and sIgA

Trace plots for the regression coefficients (*β*), residual variance (*σ²ϵ*), and pollutant-specific kernel parameters (*r*) fluctuated around stable central values without persistent trends or systematic drift, indicating satisfactory MCMC mixing and convergence for both the iNOS/Arg-1 and sIgA models (**Supplementary Figure 5** and **S6**).

**Figure 4:**
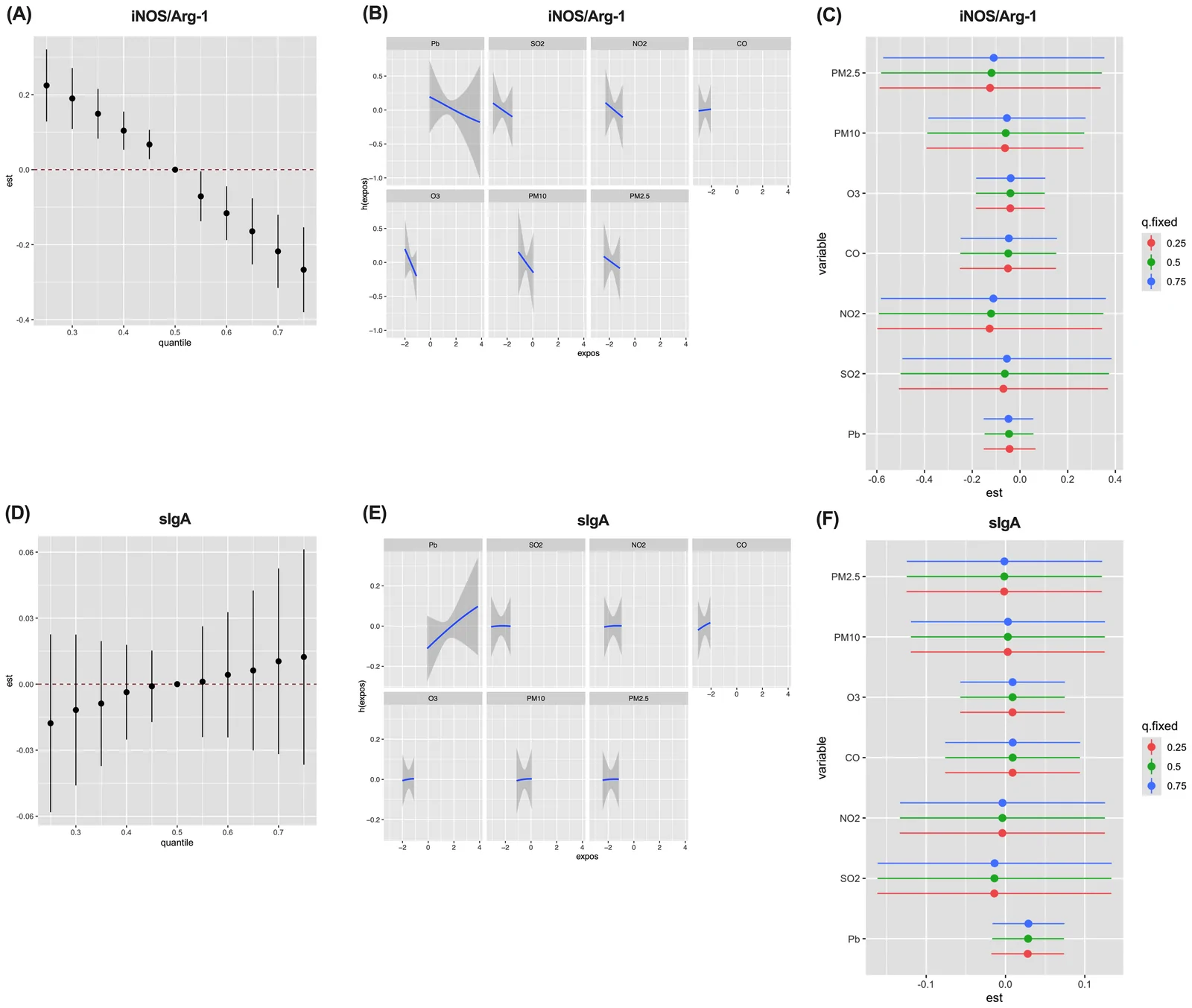
BKMR model of mixed pollutants and iNOS/Arg-1 & sIgA in children. Panels **(A–C)** show iNOS/Arg-1 results; **(D–F)** show sIgA results. **(A, D)** Single-exposure associations across quantiles. **(B, E)** Exposure-response curves. **(C, F)** Variable selection effects at different co-pollutant quantiles. All statistical models are described in Methods. All BKMR models were adjusted for sex, age, pencil/eraser biting habit, direct contact with e-waste, passive smoking, breastfeeding duration, probiotic use within the previous 3 months, air conditioning use with closed windows, and residential e-waste pollution within 50 m.

Higher levels of the overall pollutant mixture were associated with a lower iNOS/Arg-1 ratio, with the estimated mixture effect becoming progressively more negative as the joint exposure quantile increased. The corresponding 95% credible intervals remained below zero over most higher exposure quantiles (**Figure 4A**), supporting a negative overall mixture association. The univariate exposure–response functions generally showed inverse patterns for Pb and the ambient air pollutant exposure metrics, although the magnitude and uncertainty of these patterns varied across pollutants (**Figure 4B**). Single-pollutant effect estimates obtained when the remaining pollutants were fixed at the 0.25, 0.50, and 0.75 quantiles are presented in **Figure 4C**. Most estimates were negative but were accompanied by varying degrees of uncertainty. PIPs for the pollutants ranged from 0.568 to 0.756 (**Supplementary Table 2**). Taken together, the BKMR analysis indicated an inverse overall association between the pollutant mixture and the iNOS/Arg-1 ratio.

The overall pollutant mixture was not clearly associated with serum sIgA levels, and the estimated mixture effects remained close to the null across the evaluated joint exposure quantiles (**Figure 4D**). The univariate exposure–response functions showed no clear associations between most ambient air pollutant exposure metrics and sIgA, whereas Pb displayed a modest positive pattern with considerable uncertainty (**Figure 4E**). Single-pollutant effect estimates obtained when the remaining pollutants were fixed at the 0.25, 0.50, and 0.75 quantiles were generally close to the null and had wide credible intervals (**Figure 4F**). PIPs ranged from 0.196 to 0.302 (**Supplementary Table 2**). Overall, the BKMR analysis showed little evidence of an association between the pollutant mixture and serum sIgA levels.

### 3.6 Interactions among pollutants

**Figure 5:**
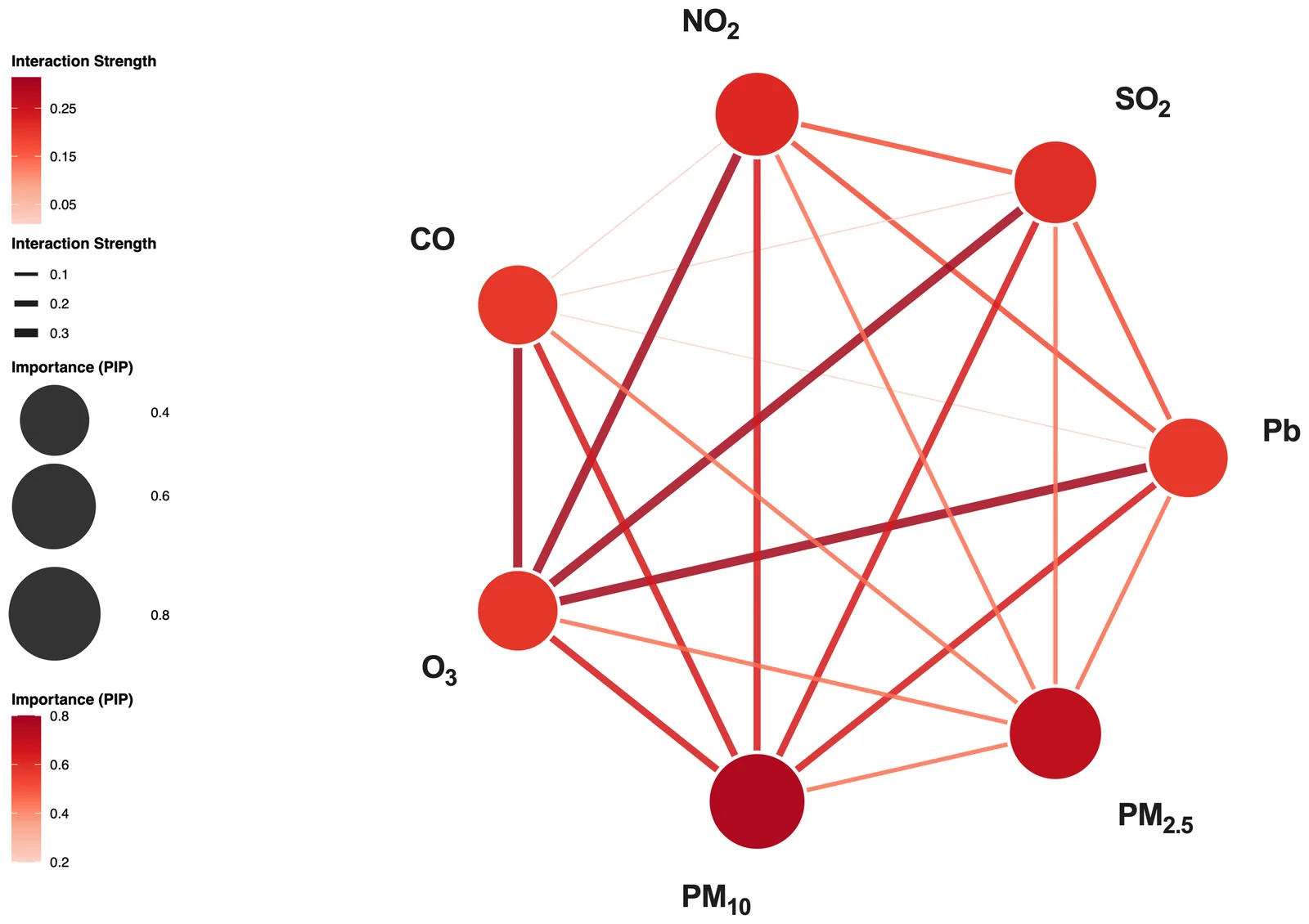
Interaction network analysis of air pollutants and Pb associated with iNOS/Arg-1. Each node represents one pollutant included in the mixture model, including SO_2_, NO_2_, CO, O_3_, PM_10_, PM_2.5_, and Pb. Node size and node color are scaled according to the posterior inclusion probability (PIP) of each pollutant, with larger and darker nodes corresponding to higher PIP values. Edges represent pairwise interaction strength between pollutants estimated from the BKMR model, with thicker and darker edges indicating stronger potential interactions.

This study further explored pairwise interaction patterns among the seven pollutants in relation to the iNOS/Arg-1 ratio using the BKMR model (**Figure 5**; **Supplementary Table 1**). The estimated interaction intensities varied across pollutant combinations. Relatively higher interaction intensity estimates were observed for Pb–O_3_ (0.3142), CO–O_3_ (0.3112), NO_2_–O_3_ (0.3109), and SO_2_–O_3_ (0.3107), whereas combinations involving CO with Pb, SO_2_, or NO_2_ showed comparatively lower estimates (0.0137–0.0185). Overall, the BKMR model suggested heterogeneous pairwise interaction patterns among pollutant combinations in relation to the iNOS/Arg-1 ratio, indicating that the estimated joint associations of pollutant pairs with this outcome differed across combinations.

### 3.7 Association between iNOS/Arg-1 and sIgA

**Figure 6:**
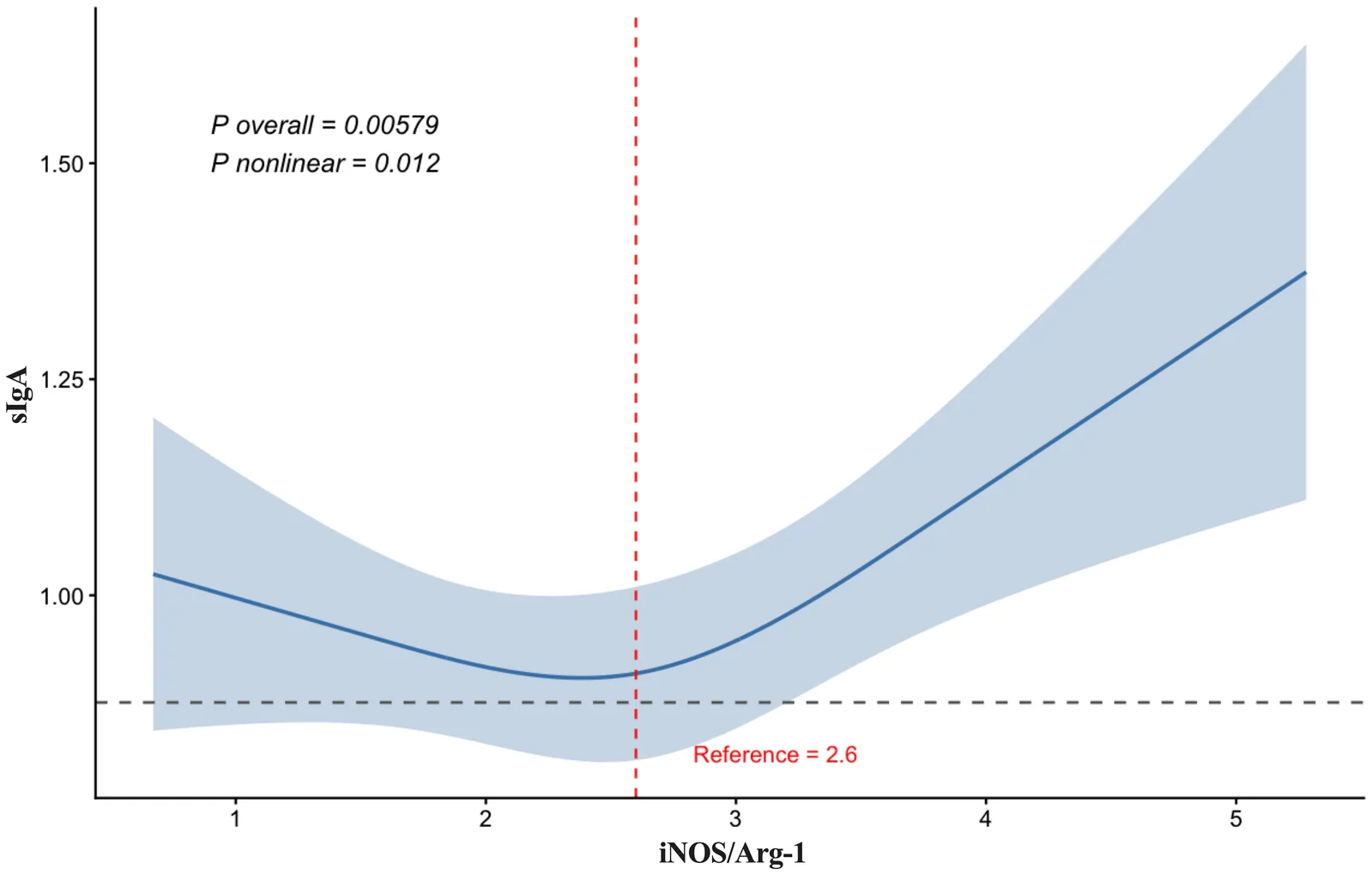
RCS analysis of the association between iNOS/Arg-1 and sIgA. The solid line represents the fitted dose–response curve, and the shaded area represents the 95% confidence interval. The vertical red dashed line indicates the reference value of the iNOS/Arg-1 ratio. *P* overall indicates the overall association, and *P* nonlinear indicates the test for nonlinearity.

RCS analysis suggested a nonlinear association between the ln-transformed iNOS/Arg-1 ratio and serum sIgA levels (*P* for nonlinearity = 0.012; **Figure 6**), with a pattern suggestive of a U-shaped relationship. The fitted curve reached its lowest point at approximately 2.6 on the ln-transformed scale, after which serum sIgA levels tended to increase with increasing ln-transformed iNOS/Arg-1 ratio.

### 3.8 Network toxicology

**Figure 7:**
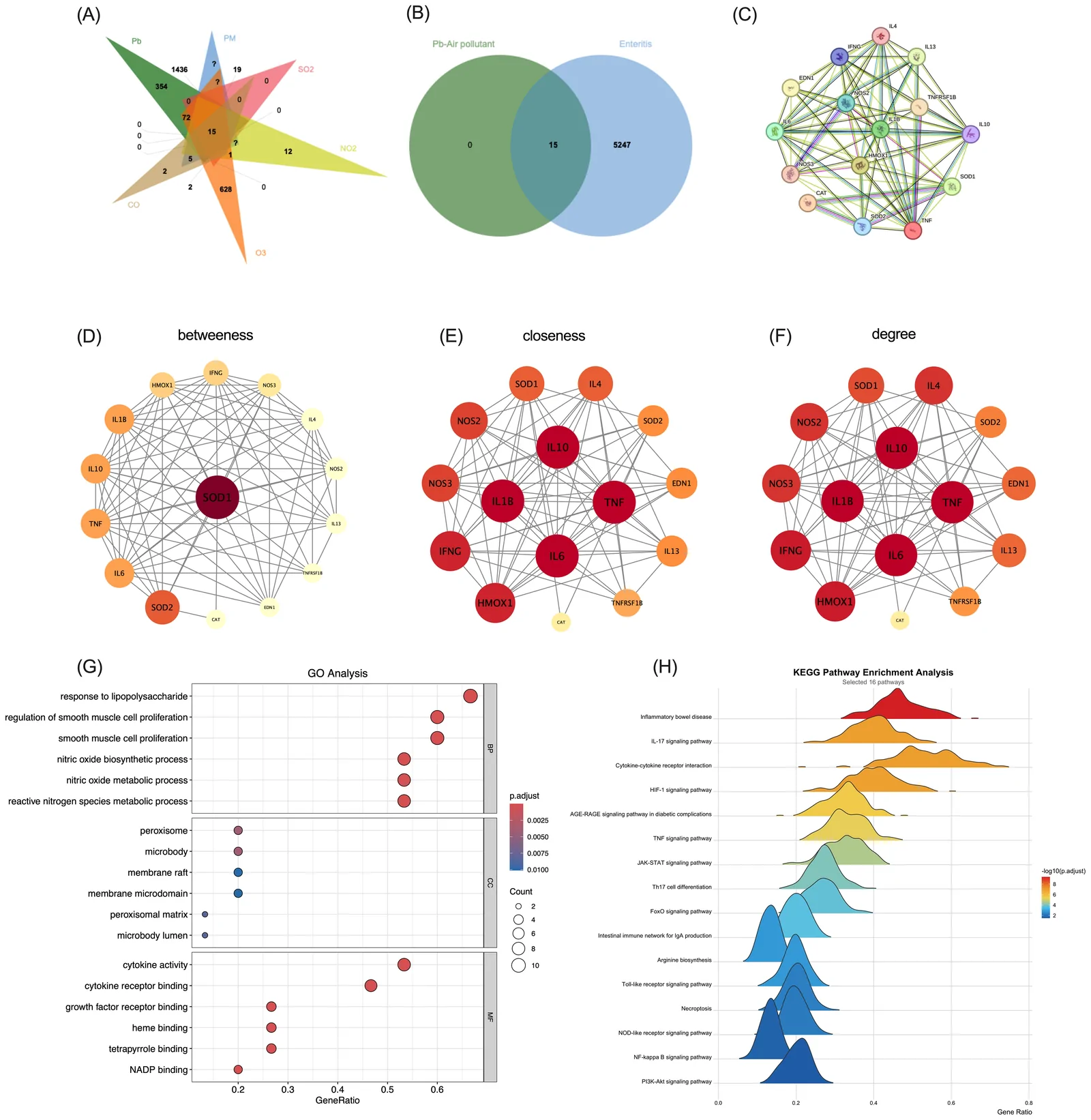
Network toxicology of the association between air pollutant and Pb exposure enteritis. **(A)** Venn diagram of air pollutant and Pb exposure-related targets. **(B)** Venn diagram of air pollutant/Pb exposure-related targets and enteritis-related targets. **(C)** PPI network of the core targets constructed using the STRING database. **(D-F)** Hub target screening based on topological analysis. **(G)** GO enrichment analysis. **(H)** KEGG pathway enrichment analysis.

Potential targets of Pb, SO_2_, NO_2_, CO, O_3_, PM_10_, and PM_2.5_ were retrieved from the CTD database, yielding 15 intersecting pollutant-related targets (**Figure 7A**). Separately, 5,262 enteritis-associated targets were obtained from the CTD and GeneCards databases. Intersection of these enteritis-related targets with the 15 pollutant-related targets identified the same 15 common targets (**Figure 7B**). A protein–protein interaction (PPI) network of these 15 core targets was constructed using the STRING database (**Figure 7C**). Hub genes were then screened using three centrality algorithms—betweenness (**Figure 7D**), closeness (**Figure 7E**), and degree (**Figure 7F**)—implemented in Cytoscape. The analysis identified SOD1, TNF, IL-6, and IL-1β as highly connected predicted targets potentially linking mixed exposure to air pollutants and Pb with enteritis-related processes.

To further explore potentially relevant biological processes and pathways, GO functional enrichment and KEGG pathway enrichment analyses were performed on the 15 predicted targets. GO analysis revealed that the targets were primarily enriched in biological processes such as response to lipopolysaccharide, reactive oxygen species metabolism, and nitric oxide biosynthesis; cellular components including membrane rafts and peroxisomes; and molecular functions such as cytokine receptor binding and growth factor receptor binding. These findings suggest that these predicted targets may be related to intestinal inflammation, oxidative stress, and mucosal barrier disruption (**Figure 7G**).

KEGG pathway enrichment analysis showed that the core targets were significantly enriched in the inflammatory bowel disease (IBD) pathway, as well as in classic pro-inflammatory signaling pathways including TNF, NF-κB, IL-17. The arginine biosynthesis pathway and intestinal immune network for IgA production were also significantly enriched (**Figure 7H**).

## 4 Discussion

Unlike our previous studies that focused primarily on the detrimental effects of environmental pollutant exposure on intestinal health (6–9), the present study investigated the association of chronic co-exposure to air pollutants and Pb with intestinal mucosal immune regulation in children, focusing on macrophage polarization-related markers and sIgA. Children from the exposed area showed lower serum iNOS, higher Arg-1 and IL-4, and a reduced iNOS/Arg-1 ratio. In addition, the iNOS/Arg-1 ratio was nonlinearly associated with sIgA. Collectively, these findings suggest that sustained environmental exposure is associated with an anti-inflammatory and repair-oriented tendency in intestinal mucosal immune regulation and a potential relationship between macrophage-related immune responses and serum sIgA.

Notably, children from the exposed area had significantly lower BMI than those from the reference area, which is consistent with previous findings that e-waste-related contaminants, including Pb, PAHs, and PM2.5-bound pollutants, are associated with impaired physical growth and alterations in growth-related pathways in children (30, 32). Evidence from pediatric inflammatory diseases suggests that intestinal inflammation and immune dysregulation may interfere with normal growth and development (33). In addition, recent studies in pollutant-exposed children have reported close links among immune imbalance, gut microbiota dysbiosis, and growth impairment (10). Therefore, the lower BMI observed in Guiyu children may reflect a broader exposure-related pattern involving intestinal immune regulation and child growth.

Our previous study from the same cohort demonstrated that serum endotoxin levels in children from Guiyu were significantly elevated (8). Acute exposure to moderate-to-high doses of endotoxin drives macrophages toward an M1 phenotype and promotes the production of pro-inflammatory mediators. In contrast, sustained exposure to higher doses of endotoxin induces macrophage tolerance, leading to a shift toward M2 polarization (34). Concurrently, serum levels of GM-CSF—a key driver of M1 macrophage polarization—were reduced in Guiyu children (7, 35). Collectively, these findings provide biological context for the lower iNOS/Arg-1 ratio and predominance of M2-associated marker features observed in the exposed children.

Previous studies have shown that exposure to environmental pollutants typically triggers a pro-inflammatory M1 macrophage response. For instance, particulate matter exposure has been shown to promote M1 polarization in alveolar macrophages (36–38). Similarly, Pb exposure induces M1 polarization of microglia—the resident macrophages of the central nervous system (CNS)—via oxidative stress pathways (39–41). In contrast, chronically exposed children in the present study exhibited lower iNOS, higher Arg-1 and IL-4, and a reduced iNOS/Arg-1 ratio, suggesting a more anti-inflammatory and repair-associated immune profile. This apparent discrepancy may be related to differences in exposure duration, dose, target tissue, and developmental stage.

Experimental evidence supports the concept that macrophage polarization may change dynamically during prolonged exposure. Li, Cai (20) reported that prolonged exposure of mice to fine particulate matter induces a time-dependent shift of peritoneal macrophages from M1 to M2, characterized by upregulation of Arg-1 in the absence of NOS2 (iNOS) expression. Short-term exposure is often associated with M1-dominant inflammatory responses, whereas prolonged exposure conditions may favor M2-associated anti-inflammatory and tissue-repair features (34). These experimental findings provide biological context for the M2-associated marker profile observed in the present study.

From a functional perspective, an M2-associated immune profile may have implications for host defense. Ma, Song (42) demonstrated that the duration of environmental PM_2.5_ exposure critically shapes host resistance to pathogens. Short-term exposure enhanced IL-6 and IFN-β secretion in influenza-infected mice and improved survival, whereas long-term exposure reduced the ability of pulmonary macrophages to secrete these cytokines, leading to increased mortality following influenza infection. These experimental findings collectively provide mechanistic context for the present observation. The M2-like profile observed in exposed children may help limit excessive inflammation and tissue injury under chronic low-level pollutant exposure. However, such a potentially compensatory immune pattern may also be accompanied by impaired antimicrobial capacity. Although M2-polarized macrophages can suppress overactive inflammatory responses, they generally have reduced pathogen clearance capacity. The downregulation of iNOS observed in the present study may therefore compromise antimicrobial function (43, 44). Consequently, under long-term environmental exposure, children may become more susceptible to intestinal infections, which is consistent with our clinical observation of a significantly higher frequency of diarrhea in Guiyu children.

The nonlinear association between the iNOS/Arg-1 ratio and serum sIgA observed in the RCS analysis may indicate a potential relationship between macrophage polarization-related immune responses and sIgA-associated mucosal immunity. One possible explanation is that variations in macrophage polarization-related markers across the observed range may be accompanied by corresponding changes in serum sIgA levels, resulting in relatively small differences in mean serum sIgA concentrations between the two groups. Notably, mixed pollutant exposure showed no significant association with serum sIgA levels, whereas a nonlinear association was identified between the iNOS/Arg-1 ratio and serum sIgA. These findings suggest that macrophage polarization-related immune alterations may be more closely associated with variation in serum sIgA than direct associations with pollutant exposure. This interpretation is biologically plausible because innate and adaptive immune responses are functionally interconnected through cytokine signaling and antigen presentation (45, 46). Collectively, these findings highlight the importance of considering the overall relationship between innate and adaptive immune responses when evaluating pollutant-associated alterations in mucosal immunity, rather than relying on any single immune indicator.

The network toxicology analysis suggested potential pathways linking pollutant exposure to intestinal immune alterations. In particular, the enrichment of pathways related to nitric oxide biosynthesis, arginine metabolism, and intestinal IgA production suggests that exposure to Pb and air pollutants may be associated with the regulation of intestinal mucosal immunity. Arginine metabolism is closely involved in macrophage polarization, with competitive arginine utilization by iNOS and Arg-1 contributing to M1- and M2-associated functional states (47). Importantly, these pathway-level findings are consistent with our previous gut microbiome and metabolomics study in children from the same e-waste setting, which showed Pb-associated alterations in gut microbial diversity, fecal microbiota, metabolites, and arginine-related metabolic pathways (9). This consistency across independent analytical approaches strengthens the biological plausibility of the present findings and provides complementary evidence supporting a potential role of gut microbial and metabolic disturbances in pollutant-associated intestinal mucosal immune regulation.

Several limitations should be acknowledged. First, the cross-sectional design precludes causal and temporal inference, and longitudinal studies are needed to determine whether the observed macrophage polarization pattern changes with exposure duration. Second, the interpretation of an M2-associated profile remains preliminary because the absolute changes in iNOS and Arg-1 were modest and no cell-specific or gene-expression data were available. Third, the nonlinear association between the iNOS/Arg-1 ratio and serum sIgA remains exploratory and requires further investigation. Although serum sIgA may reflect mucosal immune activity, serum measurements alone cannot identify the specific mucosal tissue of origin. Future studies incorporating fecal sIgA or intestinal mucosal lavage samples are warranted to validate these findings. Fourth, network toxicology findings were predictive and require experimental validation. Finally, because ambient air-pollutant exposure largely reflected the environmental contrast between the Guiyu and Haojiang cohorts, the observed air-pollutant associations cannot be readily separated from other regional characteristics and should therefore be interpreted within the broader regional context. Future studies incorporating individual-level exposure assessment, multicenter populations, and locally derived mucosal biomarkers are warranted to better distinguish pollutant-specific effects from regional contextual influences.

## 5 Conclusion

In summary, long-term co-exposure to air pollutants and Pb was associated with an anti-inflammatory and repair-oriented tendency in children’s intestinal mucosal immune regulation. This pattern was characterized by reduced iNOS, increased Arg-1 and IL-4, and a lower iNOS/Arg-1 ratio, suggesting a potential tendency toward M2 macrophage polarization. Moreover, the nonlinear association between the iNOS/Arg-1 ratio and serum sIgA suggests a potential relationship between macrophage polarization-related immune responses and sIgA-associated mucosal immunity. Collectively, these results provide immune-related evidence of pollutant-associated alterations relevant to intestinal mucosal defense and may help inform the assessment of intestinal health risks in environmentally polluted areas.

## Data Availability

The raw data supporting the conclusions of this article will be made available by the authors, without undue reservation.
