# Supplementary material for: iNOS/Arg-1 balance and sIgA levels are associated with intestinal mucosal immune defense in children chronically exposed to air pollutants and lead
Source: Front Immunol. 2026 Aug 28;17:1903043. doi: 10.3389/fimmu.2026.1903043 (PMC13561977; doi:10.3389/fimmu.2026.1903043)
Supplement: Supplementary file 1 [file Table1.docx]

**Supplementary Table S1. Bivariate pollutant interaction analysis of air pollutants and Pb exposure on iNOS/Arg-1**

|  | Pb | SO_2_ | NO_2_ | CO | O_3_ | PM_10_ | PM_2.5_ |
| --- | --- | --- | --- | --- | --- | --- | --- |
| Pb | 0 | 0.1678 | 0.1724 | 0.0137 | 0.3142 | 0.238 | 0.1389 |
| SO_2_ | 0.1678 | 0 | 0.1664 | 0.0185 | 0.3107 | 0.2379 | 0.1354 |
| NO_2_ | 0.1724 | 0.1664 | 0 | 0.0185 | 0.3109 | 0.2381 | 0.1356 |
| CO | 0.0137 | 0.0185 | 0.0185 | 0 | 0.3112 | 0.2385 | 0.1358 |
| O_3_ | 0.3142 | 0.3107 | 0.3109 | 0.3112 | 0 | 0.2381 | 0.1356 |
| PM_10_ | 0.238 | 0.2379 | 0.2381 | 0.2385 | 0.2381 | 0 | 0.1353 |
| PM_2.5_ | 0.1389 | 0.1354 | 0.1356 | 0.1358 | 0.1356 | 0.1353 | 0 |

**Supplementary Table S2. Posterior inclusion probabilities derived from the BKMR models for the iNOS/Arg-1 ratio and serum sIgA.**

|  | **iNOS/Arg-1** | **sIgA** |
| --- | --- | --- |
| Pb | 0.568 | 0.196 |
| SO_2_ | 0.598 | 0.214 |
| NO_2_ | 0.612 | 0.302 |
| CO | 0.572 | 0.256 |
| O_3_ | 0.576 | 0.244 |
| PM_10_ | 0.756 | 0.301 |
| PM_2.5_ | 0.708 | 0.274 |

**Notes:** Posterior inclusion probability (PIP) represents the posterior probability that an exposure is included in the Bayesian kernel machine regression (BKMR) model. Pb, blood lead; SO_2_, sulfur dioxide; NO_2_, nitrogen dioxide; CO, carbon monoxide; O_3_, ozone; PM_10_, particulate matter with an aerodynamic diameter ≤10 μm; PM_2.5_, particulate matter with an aerodynamic diameter ≤2.5 μm; iNOS/Arg-1 ratio, the ratio of inducible nitric oxide synthase to arginase-1; sIgA, secretory immunoglobulin A.

**Supplementary Table S3. Comparison of primary and Guiyu-restricted sensitivity analyses for blood Pb**

| **Exposure** | **Outcome** | **Analysis population** | **n** | ***β (95% CI)*** | ***P*-value** |
| --- | --- | --- | --- | --- | --- |
| Blood Pb | iNOS/Arg-1 | Full sample | 238 | −0.110 (−0.235, 0.015) | 0.086 |
| Blood Pb | iNOS/Arg-1 | Guiyu | 121 | −0.089 (−0.277, 0.099) | 0.356 |
| Blood Pb | sIgA | Full sample | 238 | 0.093 (−0.036, 0.222) | 0.160 |
| Blood Pb | sIgA | Guiyu | 121 | 0.110 (−0.077, 0.297) | 0.251 |

**Note:** Pb was natural log transformed before regression analysis. *β* values are standardized regression coefficients. Models were adjusted for sex, age, direct contact with e-waste, passive smoking, breastfeeding duration, probiotic use within the previous 3 months, air conditioning use with closed windows, and residential e-waste pollution within 50 m. The Guiyu-only analysis was conducted to reduce potential confounding from between-area differences.

**Supplementary Figure S1. Flowchart of participant recruitment and selection. A total of 680 children from kindergartens in Guiyu and Haojiang completed the questionnaire survey. Among them, 97 declined participations in the health examination, leaving 583 children who completed the physical examination and biological sample collection. After excluding 129 children because of incomplete key information, current inflammation, illness within the previous month, antibiotic use within the previous 3 months, or residence in the study area for less than one year, 454 children met the eligibility criteria. Following age- and sex-matching, 238 children were selected for laboratory testing and included in the final analysis, including 121 children from Guiyu and 117 from Haojiang.**

**Supplementary Figure S2. Directed acyclic graph for covariate selection. The directed acyclic graph illustrates the hypothesized relationships among environmental pollutant exposure, circulating immune markers, potential confounders, exposure-related factors, and downstream health or developmental variables.**

**Supplementary Figure S3. RCS analysis of dose-response relationships between air pollutant and Pb exposures and iNOS/Arg-1.**

**Supplementary Figure S4. RCS analysis of dose-response relationships between air pollutant and Pb exposures and sIgA.**

**Supplementary Figure S5. MCMC trace plots for the BKMR model evaluating the association between the pollutant mixture and the iNOS/Arg-1 ratio. (A) Trace plot of the regression coefficient (β); (B) trace plot of the residual variance (σ²ε); and (C) trace plot of the pollutant-specific kernel parameter (r). The stable oscillation of the Markov chains around constant central values without systematic trends indicates satisfactory mixing and convergence of the MCMC algorithm.**

**Supplementary Figure S6. MCMC trace plots for the BKMR model evaluating the association between the pollutant mixture and serum sIgA. (A) Trace plot of the regression coefficient (β); (B) trace plot of the residual variance (σ²ε); and (C) trace plot of the pollutant-specific kernel parameter (r). The Markov chains fluctuated randomly around stable central values with no evidence of systematic drift, indicating good convergence and reliable posterior estimation.**
